# Residency training for minimally invasive surgery

**DOI:** 10.1590/0100-6991e-20213040

**Published:** 2022-02-18

**Authors:** MARCELO ESTEVES CHAVES CAMPOS, MARILENE VALE DE CASTRO MONTEIRO, FABIANA MARIA KAKEHASI

**Affiliations:** 1 - Hospital das Clínicas da Universidade Federal de Minas Gerais, Centro de Treinamento e Educação Cirúrgica - Belo Horizonte - MG - Brasil

**Keywords:** Simulation Training, Clinical Competence, Laparoscopy, Coronavirus, Education, Medical, Treinamento por Simulação, Competência Clínica, Laparoscopia, Coronavirus, Educação Baseada em Competências

## Abstract

**Objective:**

to develop a training program in minimally invasive surgery, based on simulation and with an emphasis on the acquisition of laparoscopic competences.

**Methods:**

this was a prospective, observational study carried out at a university hospital in Belo Horizonte, Brazil, between April 2020 and January 2021. We recruited residents of surgical specialties for structured, progressive training according to instructional principles to promote learning, such as motivation, activation, demonstration, application, and integration. We filmed the skill tests at the program’s beginning, middle, and end, which were then anonymously evaluated by a surgical education expert. Individual performances were scored using the global assessment tools “GOALS” and “specific checklist for suture”. At the end, all participants received individual feedback and completed a questionnaire to assess the impact of training on the Kirkpatrick model.

**Results:**

43 residents completed the program. The evolution of performances was evident and grew between tests. The average achievements were 29% in the initial test, 43% in the intermediate test, and 88% in the final test, with significant differences between all mean scores, with H=97.59, GL=2, p<0.0001. The program evaluation and learning perceptions were excellent, but only 10.7% of residents felt fully capable of performing unsupervised, low-complexity laparoscopic surgery at the end of training.

**Conclusions:**

the training program developed in this study proved to be feasible and promising as a strategy for teaching laparoscopic surgery*.*

## RESUMO

**Objetivo:**

desenvolver programa de treinamento em cirurgia minimamente invasiva, baseado em simulação e com ênfase na aquisição de competências laparoscópicas.

**Métodos:**

trata-se de estudo prospectivo observacional que foi realizado em hospital universitário de Belo Horizonte, Brasil, entre abril de 2020 e janeiro de 2021. Foram recrutados residentes de clínicas cirúrgicas para treinamento progressivo estruturado de acordo com princípios instrucionais de promoção da aprendizagem, como: motivação, ativação, demonstração, aplicação e integração. Testes de habilidades foram filmados no início, meio e final do programa, e, então, avaliados em anonimato por perito em educação cirúrgica. As performances individuais foram pontuadas por meio das ferramentas de avaliação global “*GOALS*” e “checklist específico de sutura”. Ao final, todos participantes receberam feedbacks individuais e preencheram questionário destinado a avaliar o impacto do treinamento, baseado no modelo de Kirkpatrick.

**Resultados:**

43 residentes concluíram o treinamento. A evolução das performances foi crescente e evidente entre os testes. Os aproveitamentos médios foram: 29% no teste inicial; 43% no teste intermediário; e 88% no teste final, com diferenças significativas entre todas as médias de pontuação, sendo H=97,59; GL=2; p<0,0001. A avaliação do programa e percepções de aprendizagem foram excelentes, mas apenas 10,7% dos residentes sentiram-se totalmente capazes a realizar cirurgia laparoscópica de baixa complexidade sem supervisão ao final do treinamento.

**Conclusões:**

o programa de treinamento desenvolvido nesse estudo mostrou-se factível e promissor como estratégia de ensino da cirurgia laparoscópica.

## INTRODUCTION

At the end of 2019, a new infectious disease called coronavirus disease 19 (COVID-19) was responsible for the onset of a pandemic that affected and continues to affect health systems worldwide in different ways. There was a sudden increase in the demand for care and hospital beds, which overloaded these systems^1^. Thus, the effective functioning of essential health services was threatened due to the lower availability of resources (material, economic, and human) that were directed towards fighting the pandemic^2^. In moments of greatest upsurge of the pandemic, world health authorities recommended that elective operations be avoided, to reduce the care burden caused by the new coronavirus by reducing the exposure of medical teams and patients to potential contamination^2^ ^,^ ^3^. Such recommendations also directly impacted the teaching and training of new medical professionals, especially those inserted in medical residency programs in surgical specialties^4^ ^-^ ^6^.

Since the volume of operations in hospitals was drastically reduced, surgical education based on the Halstedian model was also compromised^7^. Therefore, in this pandemic scenario, together with the steep learning curve in the acquisition of operative skills in laparoscopy, training of residents outside the operating room was even more recommended^8^. Teaching through simulation models develops familiarity with the handling of laparoscopic instruments, as well as cognitive and technical competences in minimally invasive operations^9^.

Specific training programs in minimally invasive operations are developed and discussed in the literature^10^. However, most of these programs emphasize skill acquisition rather than competency development. To fill this gap, the objective of this study was to develop a training program in minimally invasive surgery, based on simulation and with emphasis on the acquisition of laparoscopic competences.

## METHODS

This study, which was approved by the Ethics in Research Committee (COEP Opinion No. CAAE: 0364.0.203.000-11), was conducted in a university hospital in Belo Horizonte, state of Minas Gerais, Brazil, between April 2020 and January 2021. All participants provided a written consent to participate in the research.

Initially, we developed a training program in minimally invasive surgery, with emphasis on the acquisition of laparoscopic competences and based on instructional principles to promote learning. This involved various teaching strategies, feedback, repetition of practice, variation of difficulties, increasing complexity, distributed practice, cognitive interaction, individualization of learning, clinical context variation, and knowledge integration^11^. Afterwards, we invited residents of surgical specialties to carry out the activities, which we divided into two stages. The first stage was an online module and the second consisted of face-to-face modules (Basic module and Endosuture module), corresponding to a total workload of 40 hours, distributed in 20 hours each. 

The first stage of the program (online module) occurred during the first half of 2020, a period of social isolation imposed by health authorities to stop the advance of COVID-19, which prevented classroom activities for training skills. At this stage, all residents of surgical specialties were instructed to individually attend online training on the fundamental aspects of minimally invasive operations. This training consisted of 10 weekly classes taught by specialists with extensive experience in the area on basic topics of laparoscopic surgery, such as energy used, postoperative care, abdominal synthesis, endosuture, and training models.

**Figure 1:**
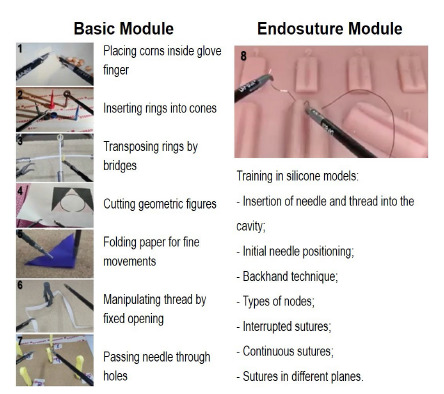
Exercises for training specific laparoscopic operative skills in dry-lab simulators. Basic Module: exercises 1 to 7; Endosuture Module: exercise 8, with 7 activities.

In the second stage, we selected 50 residents to answer a questionnaire that addressed age, gender, specialty, year of entry into the residency program, experience in laparoscopy, previous training in laparoscopy courses. The participants were residents preferably from the last year of medical residency programs in surgical specialties with a greater volume of laparoscopic surgeries. Next, we invited the participants to take the face-to-face modules, which became viable after the end of the confinement, in the second half of 2020, maintaining all the precautions recommended for the prevention of COVID-19, such as social distancing, use of masks, and frequent cleaning with alcohol. These modules consisted of review of theorical issues, demonstration of videos of operations and situations requiring certain laparoscopic skills, and application of new knowledge in training laparoscopic practice with dry Lab simulators. This stage of the program was based on simulation and proficiency, with individualized and progressive training (the resident only advanced to the next exercise after reaching the goal of the previous exercise), in addition to the presence of a tutor to assist the learner in time of need (Just In Time information)^12^. The face-to-face modules (Basic and Endosuture, sequentially) were composed of eight exercises for the development of laparoscopic skills and the practices were distributed over two days, allowing a large number of repetitions (Figure 1).

All participants received standardized explanation of suture exercise to be conducted through laparoscopy in the dry-lab simulator at three different times: before the Basic module (Test 1), after the Basic module and before the Endosuture model (Test 2), and at the end of the Endosuture module (Test 3). We filmed these tests, and the videos were anonymously viewed by a surgical education expert. Then, the participants’ performances were measured by summing up the scores of the tools Global Operative Assessment of Laparoscopic Skills (GOALS) and Specific Checklist for Suture to assess learning and allow for individualized feedback^13.14^. With the software BioEstat version 5.0, we compared the performances of residents in three different moments using the nonparametric Kruskal Wallis (H) statistical test and Dunn’s method. We considered a p<0.05 as significant.

At the end, each participant received individual feedback, in written and oral form, by a single specialist who emphasized the evolution of performance, the successes, and the skills that needed further training. Based on the Kirkpatrick’s model of four levels^15^, all received a questionnaire designed to evaluate the training program and complete a performance self-assessment, with parameters scored on a five-point Likert scale. All residents were encouraged to integrate the new knowledge and skills with real situations as elective laparoscopic operations were resumed at the hospital routine.

## RESULTS

In the online module, there were 67 registered participants. In the face-to-face modules, of the 50 residents invited, 43 (86%) completed the laparoscopic training. Of the seven residents who did not participate in this second stage, three (6%) were infected with the new coronavirus and four (80%) did not respond to the invitation. Those with COVID-19 were trained later, after disease resolution, but were not included in the study. Table 1 shows the participants’ demographic profile.

**Table 1:** Demographic profile of training participants, description of residents’ specialties, previous experience with the skill worked, and behavioral data.

Number of participants	43 residents					
Mean Age	28.5 years					
Sex	Women: 56%		Men: 44%			
Specialty and year of residency	Gynecology: 34.9% (67% R3 and 33% R2)	Urology: 20.9% (33% R3, 33% R2 and 33% R1)	General Surgery: 27.9% (75% R2 and 25% R1)	Thoracic Surgery: 4.7% (50% R2 and 50% R1)	Device Cir.: 6.9% Digestive (33% R2 and 66% R1)	Proctology: 4.7% (50% R3 and 50% R1)
Laparoscopy experience	None: 20.8%	1-10 cases/year: 49.1% 10-30 cases/year: 22.6% >30 cases/year: 7.5%				
Previous training in laparoscopy courses	None: 75.5%	Virtual simulation: 5.7% Simulation in inanimate models: 17% Simulation in animal models: 7.5%				

Legend: R3: third year resident; R2: second year resident; R1: first year resident. Note: Number of years of residency by specialty: Gynecology: 3 years; Urology: 3 years; General Surgery: 2 years (1 extra year at choice); Thoracic Surgery: 2 years; Digestive System Surgery: 2 years; Proctology: 2 years (1 extra year at choice).

**Figure 2:**
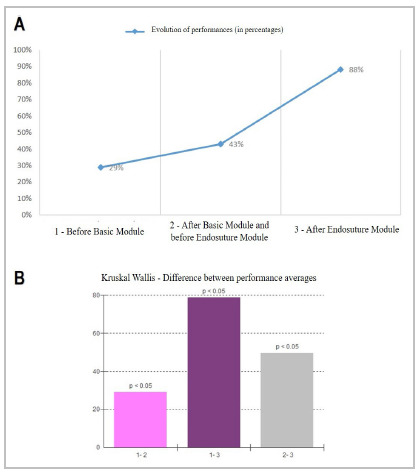
Evolution of residents' performance during training by the difference in scores between diagnostic tests in three moments. A. evolution of performances (average scores) during training; B. differences between the average performance scores, 1: being the test before the Basic module. 2: the test after the Basic module and before the Endosuture module. 3: the final test, after the end of the Endosuture module.

The percentage averages and standard deviations (DP) of the residents’ performance scores were, respectively, 29% (SD 3.4) in Test 1 (initial), 43% (SD 4.3) in Test 2 (intermediate), and 88% (SD 2.9) in Test 3 (final). The performance improvement was evident during training (Figure 2A), with significant differences between all the average scores, being H=97.59; GL=2; p<0.0001 (Figure 2B).

In the self-assessment, residents considered the levels of knowledge/skills in laparoscopy before training as poor (55.4%), fair (32.1%), and good (12.5%). After training, self-assessments of knowledge/skills in laparoscopy changed to poor (1.8%), fair (3.6%), good (51.8%), very good (41.1%), and excellent (1.8%). Despite the general progression in learning perceptions, only 10.7% completely agreed that, after training, they became capable of carrying out a low complexity laparoscopic procedure, while 75% partially agreed, 10.7% partially disagreed, and 3.6% totally disagreed. We also observed that 78.6% and 92.9% fully approved the dedicated time and training format, respectively, while 17.9% and 5.4% partially approved. The evaluation of the basic module was excellent for 80.4% of the participants and very good for 19.6%, while the Endosuture module one was excellent for 85.7% and very good for 14.3%.

## DISCUSSION

This study demonstrates the feasibility of implementing a teaching strategy based on simulation and competences, complementing and providing continuity to training of residents in the principles of minimally invasive surgery during the pandemic. The unique moment of the pandemic brought numerous care challenges that significantly impacted the residents’ training process. Just before social isolation, the Center for Surgical Training and Education had just been inaugurated at the university hospital to provide health professionals with acquisition and development of new surgical competences, in addition to the feedback on the deficiencies identified during operative procedures in simulated environments. The training sessions for this study were carried out at this center, meeting the imposed challenges.

Many authors suggest that the most effective scenarios for competence development are those that are centered on a problem^16^. Based on these authors, Merrill (2002) elaborated five instructional principles to promote learning, which are: (a) Learning is promoted when learners are engaged in solving real-world problems; (b) Learning is promoted when existing knowledge is activated as a foundation for new knowledge; (c) Learning is promoted when new knowledge is demonstrated to the learner; (d) Learning is promoted when new knowledge is applied by the learner; and (e) Learning is promoted when new knowledge is integrated into the learner’s world^17^. Another instructional design model is the 4C/ID^12^. This model has four components which are: (1) learning task, which is the problem to be solved; (2) supportive information, which is the theory to solve the problem; (3) Just-In-Time (JIT) information, which are guidelines on how to solve the problem at the exact moment the learner needs it; and (4) part-task practice, which is the repeated training of parts of the task until the problem is solved as a whole. In a way, all these instructional principles were included in the training program of the present study.

In the first stage, virtual remote teaching was responsible for supporting and motivating information. Motivation is an active part of building knowledge. The learner is an integral part of this process and there is constant interaction between the new knowledge and the prior one. In this interaction, prior knowledge incorporates other meanings and is strengthened, and if it does not exist, it will be presented, which will allow its own interpretation of the content and attribution of meaning.

Competence is directly influenced by the context^18^. Thus, a specialist considered competent in a given operation may be considered a novice in another context. In this study residents of different specialties participated, and training was divided in Basic and Endosuture modules, in which the developed laparoscopic skills were needed in different surgical contexts. The selection preferably of residents of the last year is justified, as these will be those who will soon be on the front line of patient care. The wide variability among residents’ experiences in laparoscopy demonstrated the need for individualized training to keep them motivated. 

The second stage of the program allowed the activation of knowledge and, consequently, a cognitive framework that could be improved throughout the training program. Possible contexts were incorporated at the time of demonstration, with the discussion of videos in different situations of minimally invasive procedures. The data from this study showed that the different simulated scenarios used with progressive complexity, and mainly repetitions, allowed the increasing improvement in the performance of the participants in the laparoscopic suture tests performed at the beginning, middle, and end of practical training (29%, 43%, and 88% correct answers, respectively).

As important as teaching to the learner is feedback^19^. Therefore, we used two tools that have been validated in the literature: the Specific Checklist for Suture and the GOALS^13^ ^,^ ^14^. The checklists and global scales are used for evaluation in different contexts, including simulation, each with advantages and disadvantages. Although checklists reduce the subjectivity of evaluators, evidence suggests that they can result in information loss when compared to global scales^13^ ^,^ ^14^. Thus, the joint use of these two assessment tools contributed to results interpretation and verification.

The most used model for evaluating the impact of training is the Kirkpatrick one^15^. According to this model, the outcomes of the training program for this work were excellent and can be classified into four levels. The first level would be the reaction of the apprentice who fully approved the proposed program. The second would be the learning itself, displaying a significant improvement after training in the apprentices’ perception. The transfer of learning, with a clear evolution in the skills and knowledge, would be the third level, while in the fourth would be the performance results in real operations. These levels are traditionally presented in a hierarchical order where results depend on good results at each of the initial levels^15^.

The main limitation of the present study was the lack of training in in vivo operations. The strictly simulated training may have influenced the insecurity of some participants when questioned whether they considered themselves able to perform a laparoscopic procedure at the end of training. While simulation is fundamental to the learning process, it is certainly not a substitute for real experience. However, this training process, regardless of the current pandemic situation, must precede the surgery in the real patient and this, when performed, must be supervised until the surgeon is fully trained. Thus, in view of the results, learners became able to apply the learned content in their own practice environments after this simulated training in minimally invasive surgery. However, validation studies are needed before large-scale implementation of this program. We should note that this method of learning opens new fields of research that lead to a deeper understanding of the competences involved in training surgery residents.

## CONCLUSION

The training program developed in this study, based on simulation and with an emphasis on the development of laparoscopic competences, proved to be feasible and promising, making it an excellent strategy for teaching minimally invasive surgery to resident physicians.
